# Multicenter and multimodal imaging study reveals rare fundus lesions in patients after SARS-CoV-2 infection

**DOI:** 10.1038/s41598-024-65216-9

**Published:** 2024-06-22

**Authors:** Guangqi An, Bo Lei, Zhili Wang, Kaizhuan Yang, Dongsheng Fan, Bing Li, Ke Fu, Haixin Fang, Min Zhang, Lin Li, Yu Zhao, Xuemin Jin, Liping Du

**Affiliations:** 1 Department of Ophthalmology, The First Affiliated Hospital of Zhengzhou University, Zhengzhou, Henan China; 2 Institute of Fundus Diseases, Zhengzhou University, Zhengzhou, Henan China; 3 Henan Eye Hospital, Henan Provincial People’s Hospital, People’s Hospital of Zhengzhou University, Zhengzhou, Henan China; 4 The Second People’s Hospital of Zhengzhou, Zhengzhou, Henan China; 5 Department of Ophthalmology, Luoyang Central Hospital Affiliated to Zhengzhou University, Luoyang, Henan China; 6 Nanyang Municipal Eye Hospital, Nanyang, Henan China; 7 Department of Ophthalmology, The First Affiliated Hospital of Nanyang Medical College, Nanyang, Henan China; 8 Eye institute, Henan Academy of Innovations in Medical Science, Zhengzhou, Henan China

**Keywords:** Retina, COVID-19, Acute macular neuroretinopathy, Multimodal Imaging, SARS-CoV-2, Eye abnormalities, Retinal diseases, Vision disorders

## Abstract

To define the characteristics of fundus manifestations in patients after SARS-CoV-2 infection with multimodal imaging techniques. This is a retrospective multicenter and multimodal imaging study including 90 patients. All patients with a visual complaint occurring immediately after SARS-CoV-2 infection were referred to six clinics between December 2022 and February 2023. Demographic information and the temporal relationship between SARS-CoV-2 infection and visual symptoms were documented. The characteristics of the fundus lesions were evaluated using multimodal imaging. Ninety patients from six hospitals were included in this study, including 24 males (26.67%) and 66 (73.33%) females. Seventy-eight patients (86.66%) (146 eyes) were diagnosed with Acute Macular Neuroretinopathy (AMN). The AMN patients were primarily young women (67.95%). Sixty-eight patients (87.18%) had AMN in both eyes. Thirty-eight eyes (24.36%) included Purtscher or Purtscher-like lesions. optical coherence tomography and infrared retinal photographs can show AMN lesions well. Eleven cases were diagnosed with simple Purtscher or Purtscher-like retinopathy (2 cases, 2.22%), Vogt‒Koyanagi‒Harada (VKH) syndrome or VKH-like uveitis (3 cases, 3.33%), multiple evanescent white-dot syndrome (MEWDS) (2 cases, 2.22%), and rhino-orbital-cerebral mucormycosis (ROCM) (5 cases, 5.56%). After SARS-CoV-2 infection, diversified fundus lesions were evident in patients with visual complaints. In this report, AMN was the dominant manifestation, followed by Purtscher or Purtscher-like retinopathy, MEWDS, VKH-like uveitis, and ROCM.

## Introduction

Coronavirus disease 2019 (COVID-19), caused by severe acute respiratory syndrome coronavirus 2 (SARS-CoV-2), is a life-threatening disease with a serious respiratory infection and multiorgan involvement^1^. SARS-CoV-2 primarily affects the anterior segment of the eye. The most frequently reported ocular conditions include conjunctival hyperemia, chemosis, epiphora, and even frank conjunctivitis^2,3^. It is controversial whether SARS-CoV-2 affected the retina in the early stages of the COVID-19 pandemic^4^.

Since 2019, the SARS-COV-2 virus has undergone adaptive mutation that could lead to an increase in transmissibility and virulence or a change in the presentation of clinical disease^5^. The Delta variant was identified in December 2020, and the Omicron variant was identified in November 2021, both of which caused surges in the number of infections^6^.

With the increasing number of COVID-19 patients, a growing number of changes have been observed in the retina and choroid^7^. In 2020, a cross-sectional study revealed retinal lesions associated with SARS-CoV-2, including dilated veins (27.7%), tortuous vessels (12.9%), retinal hemorrhages (9.25%), and cotton-wool spots (7.4%)^8^. A review in 2022 suggested the potential involvement of the posterior segment in SARS-CoV-2, either in the initial or later stage^9^. However, few studies have systematically described the manifestations of the posterior segment of the eye by multimodal imaging study^10^.

Due to the lockdown policy was suddenly lifted, there was a surge in the number of Omicron infections in Chinese people from late December 2022 to late January 2023 in China^11^. Based on the large number of SARS-CoV-2 infected patients, it was possible for us to observe a series of patients with rare fundus Lesions after SARS-CoV-2 infection. These rare fundus lesions with a large increase over the same period of the previous year may be related to SARS-CoV-2 infection. Because these lesions are rare and often lead to misdiagnosis, reasonable examination methods can help us quickly identify lesions and diagnose them.

In the present case series, multimodality imaging was used to describe retinal and/or choroidal conditions that could be associated with SARS-CoV-2 infections. We outlined the features of retinal and choroidal manifestations following SARS-CoV-2 infections and clarified the best diagnostic methods to improve our understanding of their pathogenesis and diagnosis.

## Materials and methods

### Participants

This was a cross-sectional multicenter and multimodal imaging study. From December 2022 to February 2023, 90 patients were identified with retinal and/or choroidal conditions associated with COVID‑19 infection in six clinics, including the First Affiliated Hospital of Zhengzhou University and/or Zhengzhou University People’s Hospital and/or The Second People's Hospital of Zhengzhou and/or Luoyang Central Hospital Affiliated to Zhengzhou University and/or Nanyang Eye Hospital, Nanyang and/or The First Affiliated Hospital of Nanyang Medical College. The incidence of these diseases in the same period of the previous year was collected from the outpatient information databases of those hospitals.

All patients tested positive by real‑time reverse transcription‑polymerase chain reaction (RT‑PCR) and/or antigen detection for SARS‑CoV‑2 from pharyngeal or nasopharyngeal swabs during the active phase of SARS‑CoV‑2 infection and eye symptoms occurred whitin one month after SARS‑CoV‑2 infection. Patients who were not infected with SARS‑CoV‑2 and whose ocular symptoms occurred more than one month after SARS‑CoV‑2 infection were excluded.

This study was approved by the Ethics Committee of the First Affiliated Hospital of Zhengzhou University [2023-KY-0637]. Based on the *Declaration of Helsinki*, we collected demographic information, SARS‑CoV‑2 infection symptoms, chief complaints and other clinical examination results after informed consent was signed by the patients or their guardians. The clinical examination results included slit‑lamp examination and indirect ophthalmoscopy results, best corrected visual acuity (BCVA), spherical equivalent (SE), fundus photo images, visual field analysis (VF), infrared retinal photographs (IR), optical coherence tomography (OCT), optical coherence tomography angiography (OCTA), fundus fluorescein angiography (FFA), indocyanine green angiography (ICGA), multifocal electrophysiology (mf ERG), fundus autofluorescence (FAF) and adaptive optics (AO). All patients were diagnosed and recorded by 2 doctors individually. In cases of disagreement, the senior ophthalmologist made the final decision.

### Method of eye examination

All outpatients were examined by slit‑lamp and ophthalmoscopy. BCVA and SE were tested by optometrists. Fundus photos and FAF were taken using an ultrawide field fundus camera (Daytona P200T, Optomap, UK) and fundus camera (CLARUS 500 v1.1, Carl Zeiss Co. Ltd., Germany).

mf ERG (RETI-scan multifocal ERG (Roland Consult, Germany) was performed on the patients. VF examination (Humphrey Field Analyzer 3, Carl Zeiss Co. Ltd., Germany) or micro examination (MP3-microperimerter Ver.1.2.1, NIDEK Co. Ltd., Japan) was selected according to the patient's symptoms.

Skilled ophthalmologists performed swept source OCT (SS-OCT) (VG200 or VG100, SVision, China) or spectral domain OCT (SD-OCT) (Spectralis OCT, Heidelberg Engineering GmbH, Germany), which included stars in 16 or 32 lines and multiline scanning with a scan line length of 10–16 mm. SS-OCTA scanning was performed with a scan square of 6 mm × 6 mm or 12 mm × 12 mm. The depth of the scanning was 3–6 mm. The scanning laser wavelength was 1050 nm or 870 nm. IR images were taken by a 40° × 40° confocal scanning superluminescent (diode) ophthalmoscope (cSSO) with a wavelength of 820 nm or confocal scanning laser ophthalmoscope (cSLO) with a wavelength of 810 nm.

FFA and ICGA (Spectralis HRA, Heidelberg Engineering GmbH, Germany) were performed to observe vascular lesions. AO (rtx1, Imagine Eyes, Orsay, France) was performed to clarify the status of the optic cone optic rod cells.

### Statistical analysis

SPSS statistics 26.0 software (IBM Corp.; USA) were used to analyze the data. Categorical data are described using frequencies and percentages. The Kolmogorov‒Smirnov test was performed to verify whether all the data sets were distributed normally. Continuous variables with a normal distribution are presented as the mean ± standard deviation (SD), and a *t* test was used for comparisons. Nonnormal variables are reported as medians (interquartile ranges). A *P* value less than or equal to 0.05 was considered statistically significant.

## Results

### COVID-19 infection, vaccination history and demographics of patients

Ninety patients were included in this study, including 24 males (26.67%) and 66 (73.33%) females. Their age was 31 ± 15 years old with a range of 10–85 years (Table 1).

**Table 1:** COVID-19 history and demographics of patients.

Disease	Number of cases (male cases) n = 90 (34, 37.78%)	Age	Visual symptoms occurred after COVID-19 symptoms	Combined with Purtscher-like retinopathy
Acute macular neuroretinopathy	78 (22, 28.21%)	29 ± 11 yrs	0–20 days median 2 days, IQR [2, 3]	17
Multiple evanescent white-dot syndrome	2(1, 50.00%)	22 and 37 yrs. respectively	5 days	0
Vogt-Koyanagi-Harada syndrome-like uveitis	3(0, 0.00%)	34,43 and 49 yrs. respectively	4,5 and 7 days respectively	0
Rhino-orbital-cerebral mucormycosis	5(1, 20.00%)	70 ± 15	5–30 days Average 14 ± 10 days	2

All patients had a history of COVID-19 infection. Visual symptoms occurred 0–30 days after the onset of fever, dry cough, malaise, etc. The mean interval between COVID-19 infection and visual symptom onset was median 2 days, IQR [2, 3]. Five patients had diabetes, six patients had kidney disease, four patients had hypertension.

We reviewed the detailed COVID-19 infection history, medication history and vaccination history in 33 of the 90 patients. All patients had a fever of 37.5–40 °C. Among them, 24 patients (71.88%) took ibuprofen, acetaminophen or Tylenol to reduce fever, whereas 9 (28.125%) did not. Twelve patients (36.37%) were dehydrated (sweating profusely and unable to drink) prior to the onset of symptoms, while 20 patients (63.63%) were dehydrated. Seven patients (21.21%) received 2 doses of inactivated COVID-19 vaccine, 19 patients (57.58%) received 3 doses of inactivated COVID-19 vaccine, and 7 patients (21.21%) did not receive inactivated COVID-19 vaccine.

### Clinical characteristics of our case series and multimodal imaging

Among all patients, 78 (86.66%) were diagnosed with acute macular neuroretinopathy (AMN). The remaining 12 patients were diagnosed with simple Purtscher-like retinopathy (2 patients, 2.22%), Vogt‒Koyanagi‒Harada-like (VKH-like) uveitis (3 patients, 3.33%), multiple evanescent white-dot syndrome (MEWDS) (2 patients, 2.22%), and rhino-orbital-cerebral mucormycosis (ROCM) (5 patients, 5.56%).

However, in December 2021 to February 2022 year, there were only one Purtscher retinopathy patient with chest trauma, one ROCM patient and two AMN patients. Although there are as many as 21 patients first diagnosed as VKH, their clinical symptoms are different from those after SARS-CoV-2 infection. The number of MEWDS patient cannot obtain because MEWDS does not have an ICD diagnostic code.

#### Acute macular neuroretinopathy (AMN) and Purtscher or Purtscher-like retinopathy

A total of 78 patients (146 eyes) were diagnosed with AMN, including 22 males (28.21%) and 56 (71.79%) females. Their age was 29 ± 11 years old with a range of 10 to 64 years old. The majority of patients were young women (67.95%). There was no statistically significant difference in age between the sexes (*t* = − 0.22, *P* = 0.830). Their complaints were "black shadows or dark spots in front of the eyes or visual field defects" (38 cases, 48.72%) and "blurred vision. "(40 cases, 51.28%).

Ten patients (12.82%) had visual problems in one eye. Sixty-eight patients (87.18%) had visual problems in both eyes. The BCVA of the 146 diseased eyes was median 0.13, IQR [0.00–0.36] logMAR with a range of 0.00 to 2.00 Log MAR. In a total of 146 eyes with AMN, the BCVAs at presentation were generally well documented to be 0.30 LogMAR or better (96 eyes, 65.75%). 1.00 LogMAR or worse in 18 eyes (12.33%).

At the initial visit, nineteen of the seventy-eight cases (38 of 146 eyes; 24.36% of cases, 26.03% of eyes) presented with either cotton-wool spots or Purtscher-like retinopathy. Ten of these patients (52.63%) had kidney disease or hypertension.

**Figure 1:**
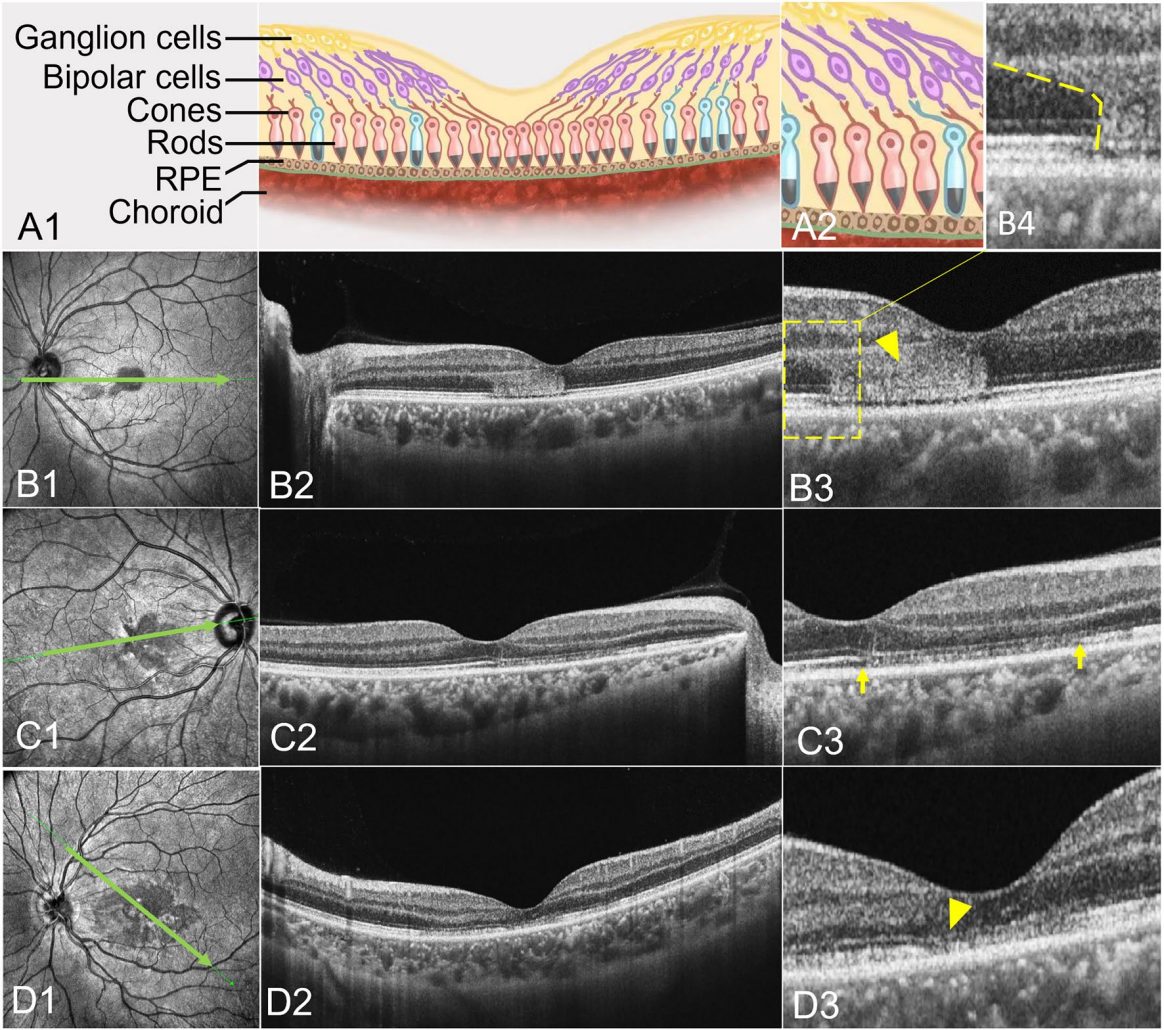
The cSSO images (a type of IR image) (**B1**; **C1**; **D1**) and the SS-OCT B-scan images (**B2**, 3,4; **C2**,3; **D2**,3) of AMN lesions. AMN lesions were dark or gray with well-demarcated margins with oval (**B1**), petal-shaped (**C1**), multifocal dark spots (**D1**). The acute phase of AMN lesions exhibited one or more abnormal characteristics, including outer retinal hyperreflectivity in SS-OCT B-scan images (**B2**, **B3**, **B4**). The shape of the lesion was consistent with the nerve fiber direction of the cone and rod cells (**A2**, **B4** yellow dotted line). The later period of AMN lesions exhibited ellipsoid zone loss (**C2**, **C3**) and thinning of the outer nuclear layer (**D2**, **D3**).

**Figure 2:**
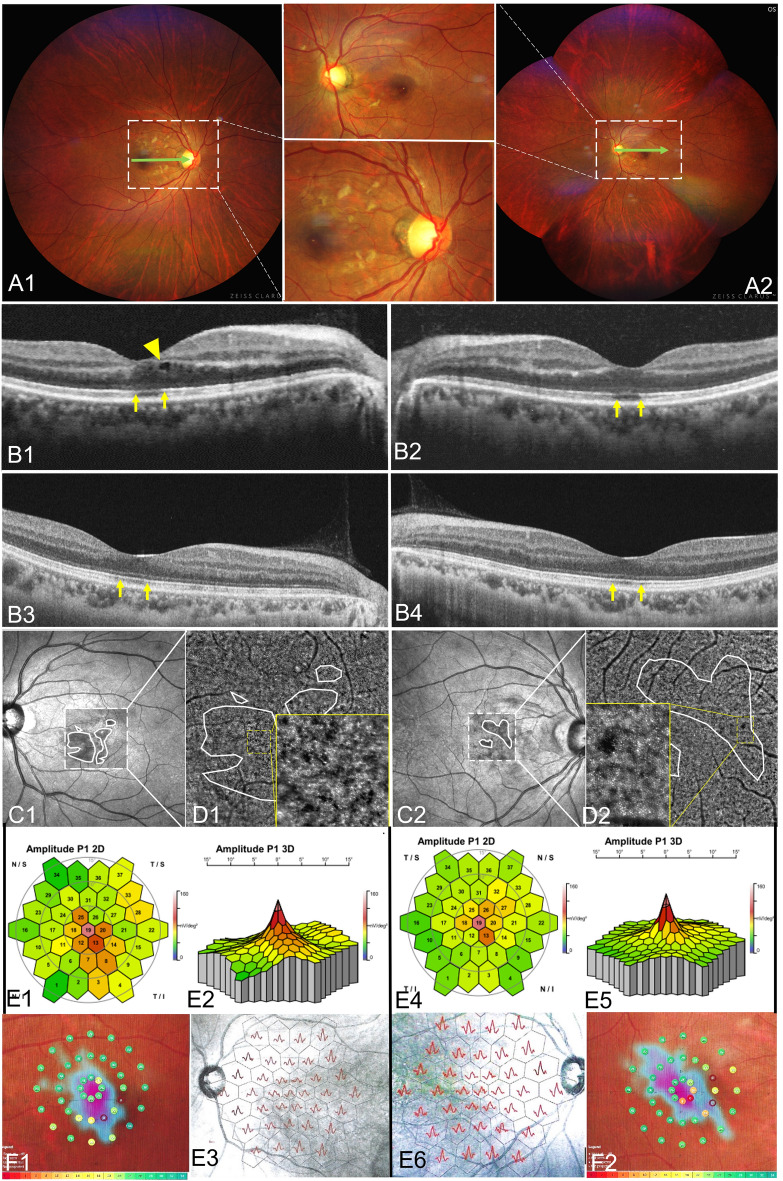
Multimodal fundus images of a 40 year-old female with AMN.Fundus photos (**A**), SS-OCT B-scan images (**B**), IR images (**C**), AO images (**D**), mf ERG (**F**) and micro-VF images (**F**). The BCVAs of her right and left eyes were 0.52 and 0.30 LogMAR (A; B1,2; F). After a month, the BCVAs of her right and left eyes increased to 0.1 and 0.00 LogMAR (B3,4; C; D; E). Compared with B1 and B2, the ellipsoid zone loss in B3 and B4 was largely restored, and the macular edema in B1 disappeared in B3.

OCT with IR were used in 146 eyes (100.00%), and in each IR image, AMN lesions were dark or gray with well-demarcated margins with oval (Fig. 1B1), petal-shaped (Fig. 1C1), multifocal dark spots (Fig. 1D1). AMN lesions on OCT exhibited one or more abnormal characteristics, including outer retinal hyperreflectivity (Fig. 1B2,B3 yellow arrowhead) which was relation to photoreceptors and bipolar cells (Fig. 1A1,A2), ellipsoid zone loss (Fig. 1C2,C3 yellow arrows), small cavity in macula (Fig. 2B1 yellow arrowhead) and thinning of the outer nuclear layer (ONL) (Fig. 1D2,D3 yellow arrowhead).

**Figure 3:**
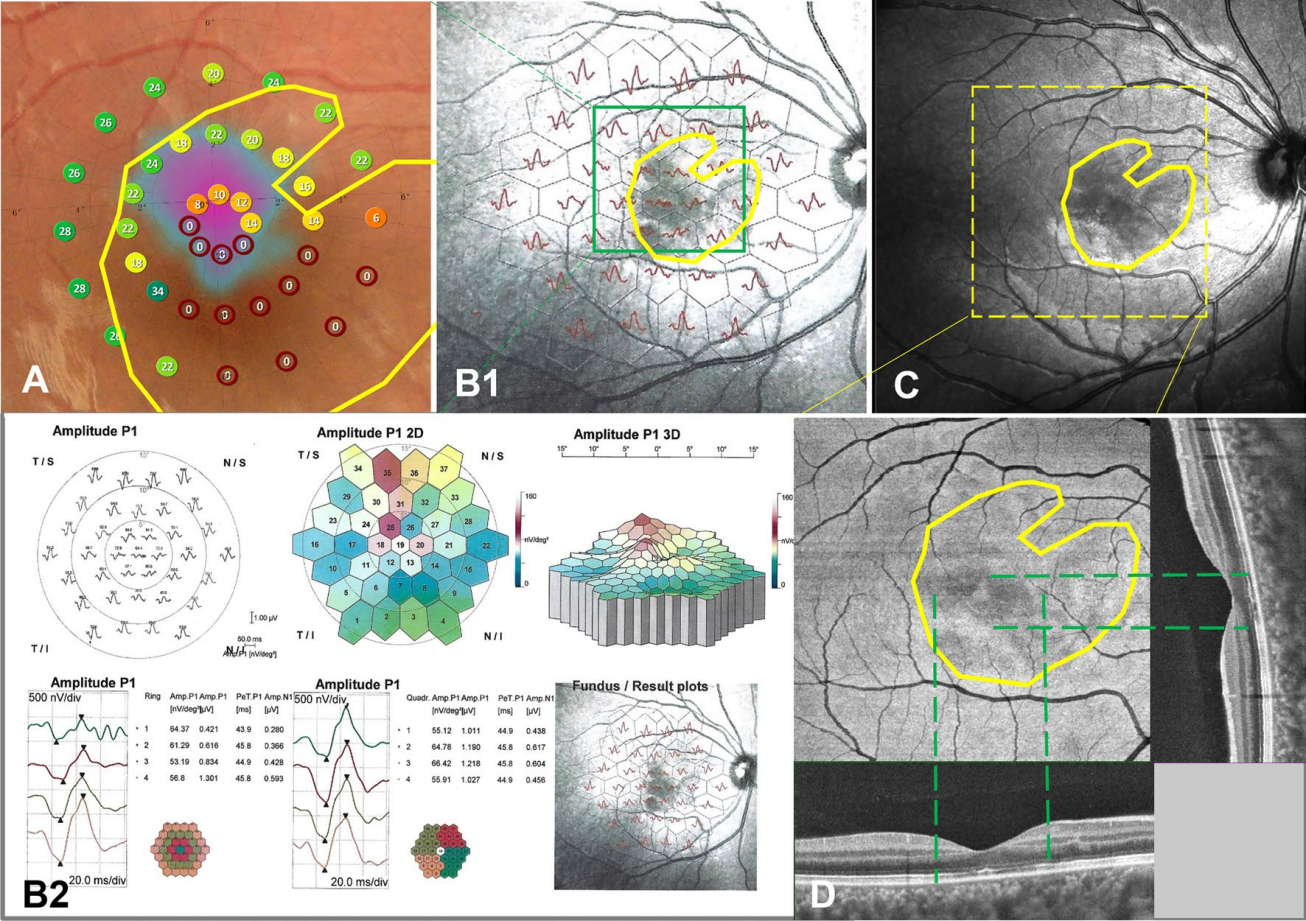
Multimodal fundus images of an 11 year-old female with AMN. Micro VF and fundus photos (**A**), mf ERG (**B1**, **B2**), IR images (**C**) and *en face* images analyzed based on SS-OCTA and SS-OCT B-scan images (**D**). The BCVAs of her right and left eyes were 0.80 and 0.70 LogMAR. The area enclosed by the yellow line shows the extent of the lesion. The dark area on the *en face* image (**D**) was consistent with the range of ellipsoid loss in the B-scan (green dashed line), which was smaller than those areas on Micro VF, mf ERG and IR images.

Fundus photos were taken in 57 of 146 eyes. The lesions showed wedge-shaped petal-shaped, oval slightly dark areas in 16 eyes (28.07%) (Fig. 2 A1,A2). The contrast sensitivity of the fundus photo was not as good as that of IR and OCT (Fig. 2B1,B2,B3,B4,C1,C2, Fig. 3C). Four eyes underwent AO and showed small patches of cone rod cell loss in the area corresponding to the lesions (Fig. 2D1,D2 yellow boxes). Ten eyes underwent mf ERG and all showed one to several abnormal findings, including diminished amplitudes and diminished implicit time (Fig. 2E1,E2,E3,E4,E5,E6, Fig. 3B1,B2). Thirty-six eyes with AMN experienced one to several paracentral scotomas by Amsler grid, VF or micro-VF testing. The mf ERG and VF specialty corresponded closely to the shape and location of the clinical lesion (Fig. 3A,B1). The shape of VF abnormalities was wedge-shaped, boot-shaped, and round-shaped (Fig. 2F1,F2). The *en face* OCT exhibits the same hypo reflectivity as the OCT B-scan (Fig. 3D).

#### VKH-like uveitis and multiple evanescent white-dot syndrome (MEWDS)

**Figure 4:**
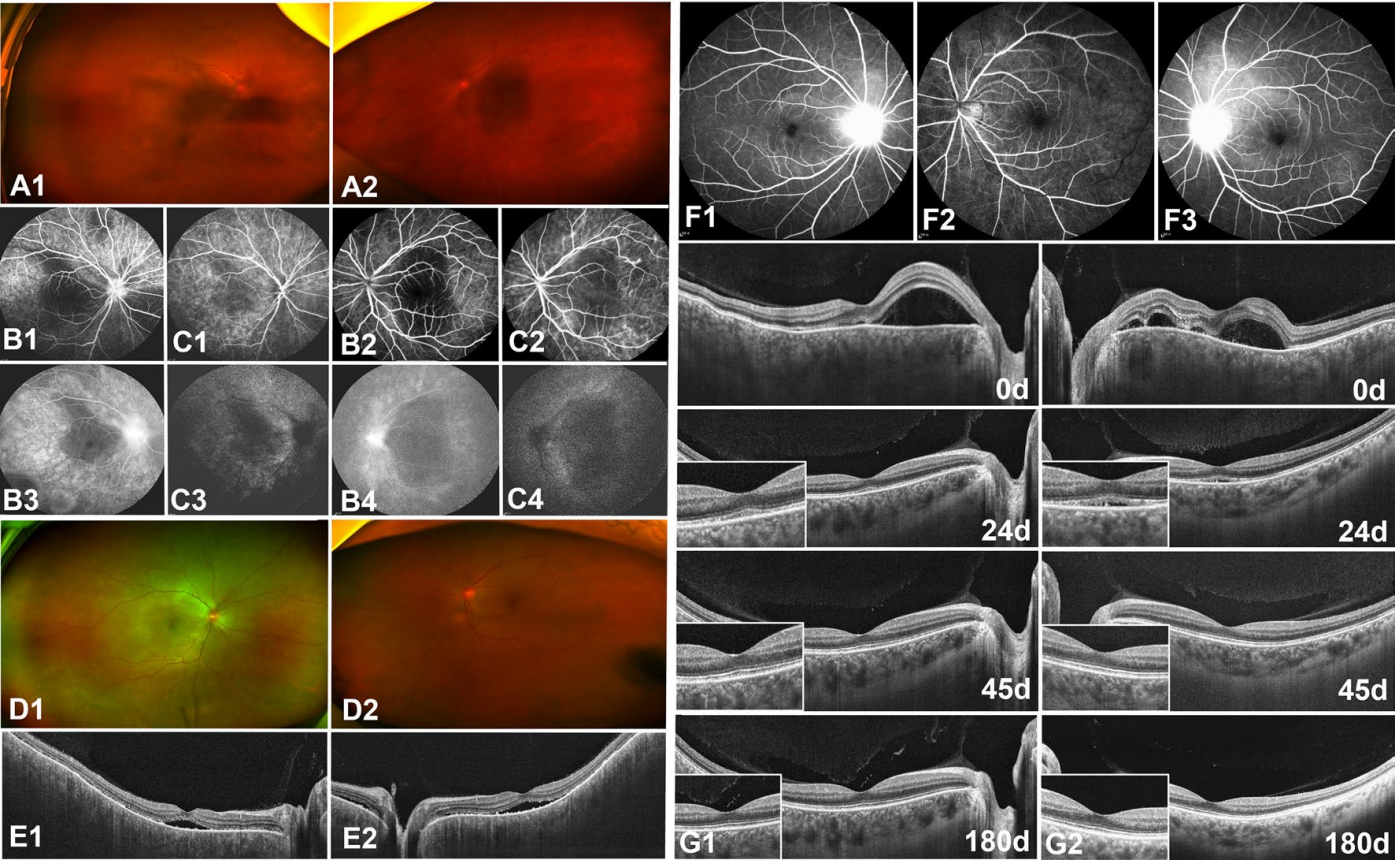
Multimodal fundus images from a 49-year-old female (**A**, **B**, **C**, **D**, **E**) and a 43 year-old female (**F**, **G**). Initially, the vitreous was opaque in the 49-year-old female (**A**). FFA (**B**) and ICGA (**C**) showed optic disc leakage and fluorescence accumulation in the advanced macula. After 3 days of treatment, the vitreous opacity was reduced (**D**), and neurosensory retinal detachment decreased (**E**). In the 43 year-old female, FFA (**F**) initially showed optic disc leakage. After treatment, neurosensory retinal detachment decreased, and choroidal thickness was reduced over time (**G**).

A 49-year-old female patient presented with complaints of visual distortion in both eyes, without accompanying headache or tinnitus (Fig. 4A,B,C). Initial BCVAs were 0.52 Log MAR for the right eye and 1.3 Log MAR for the left eye. Following the onset of fever, she was diagnosed with SARS-CoV-2 via an antigen test. Five days post-diagnosis, the patient reported progressive hazy vision in her left eye, prompting a hospital visit. A month later, similar symptoms developed in her right eye. Ophthalmic examination revealed conjunctival congestion and peripheral anterior synechia in both eyes. The vitreous was cloudy, obscuring the fundus, indicative of significant intraocular inflammation with both anterior chamber and vitreous haze initially graded at 3 + based on the Standardization of Uveitis Nomenclature (SUN) Working Group^12^ criteria with mutton-fat keratic precipitates (KPs). Therapeutic intervention was initiated with prednisone (60 mg daily), tobramycin-dexamethasone eye drops (administered every four hours), and 0.1% atropine eye ointment (applied twice daily). Significant reduction in anterior segment inflammation was observed within three days, inflammation in the right eye's anterior chamber and vitreous haze decreased to 0.5 + , while the left eye improved to 1 + , and the fundus became visible (Fig. 4D1,D2). OCT performed at this time revealed multifocal serous neurosensory retinal detachment (Fig. 4E1,E2). Subsequent improvement in BCVAs was noted, with values of 0.22 Log MAR in the right eye and 0.30 Log MAR in the left eye, alongside the resolution of neurosensory retinal detachment. The rapid amelioration of inflammation across both the anterior chamber and vitreous highlights the efficacy of the prescribed treatment regimen.

Another 43-year-old female presented with complaints of blurred vision in both eyes, without accompanying headache or tinnitus. Four days after experiencing a fever due to SARS-CoV-2 infection, her BCVAs were 0.10 LogMAR in both eyes. FFA and OCT were performed (Fig. 4F1, F2,F3,F4,G1,G2), leading to a diagnosis of VKH syndrome. Initial examination revealed anterior chamber and vitreous cells graded at 1 + with mutton-fat KPs, and vitreous haze also graded at 1 + . Treatment was initiated with prednisone (60 mg once daily) and 0.1% atropine eye ointment (one drop twice daily). The patient experienced rapid improvement; after seven days of treatment, her BCVAs improved to 0.00 LogMAR in both eyes. Subsequent FFA and OCT imaging showed a reduction and eventual resolution of neurosensory retinal detachment over time (Fig. 4G1,G2). The patient underwent a one-month course of systemic corticosteroid treatment and did not experience any relapse over the following six months.

Following SARS-CoV-2 infection, these two patients developed blurred vision and exhibited inflammation in both the anterior chamber and the vitreous body. Their symptoms are consistent with the typical manifestations of Vogt-Koyanagi-Harada (VKH) disease, characterized by pan-uveitis and multifocal serous retinal detachment, specifically granulomatous uveitis. Notably, this included mutton-fat KPs, a hallmark of VKH disease. However, unlike typical VKH cases, they did not show systemic symptoms such as tinnitus and skin changes. However, they responded well to corticosteroid treatment without any relapse.

**Figure 5:**
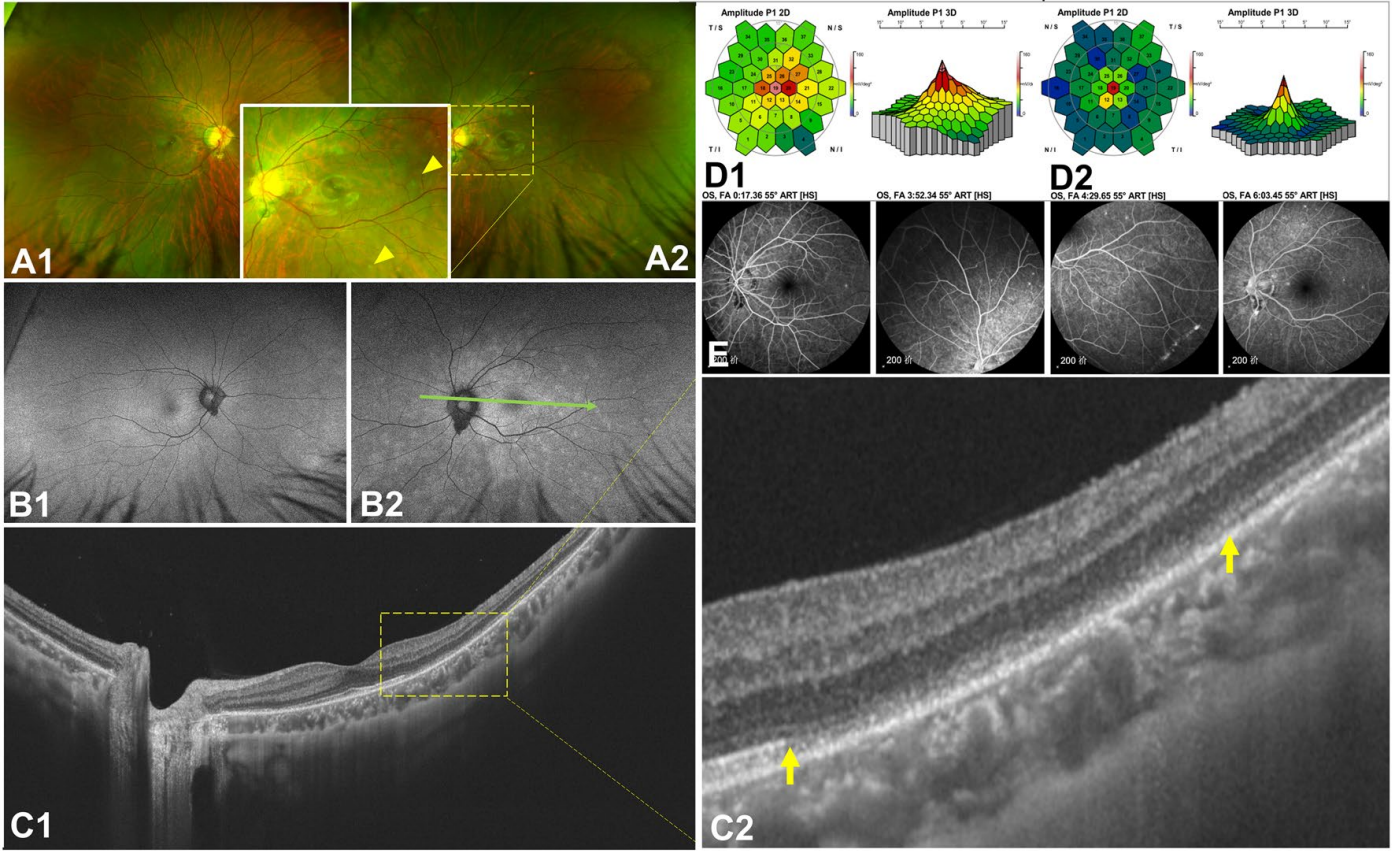
Multimodal fundus images from a 21 year-old maleFundus photos (**A**), FAF (**B**), OCT (**C**), mf ERG (**D**), and FFA (**E**). The area enclosed by the yellow line and indicated by a yellow arrowhead in A2 shows the extent of the lesion. The range of ellipsoid loss in C2 was shown (yellow arrow).

A 21-year-old male patient was diagnosed with MEWDS and complained of "blurred vision on the left eye" five days after a fever caused by SARS-CoV-2. The BCVAs were 0.00 and 0.10 LogMAR in his right and left eyes. Some yellow-white punctate lesions were faintly seen on fundus photography (Fig. 5A1,A2), OCT showed that there were structural abnormalities in the outer retina (Fig. 5C1,C2). The mf ERG showed that the visual sensitivity of the patient's left eye was reduced (Fig. 5D1,D2). There were some highly fluorescent lesions on the FAF (Fig. 5B1,B2) and scattered high fluorescence spots on FFA (Fig. 5E). However, the patient did not undergo ICGA due to drug allergies. We suggested that the patient should return to the clinic two weeks later, but he did not return. Then, we performed a telephone follow-up and were informed that his symptoms had been eliminated. Another 37-year-old female had similar symptoms.

#### Rhino-orbital-cerebral mucormycosis (ROCM)

**Figure 6:**
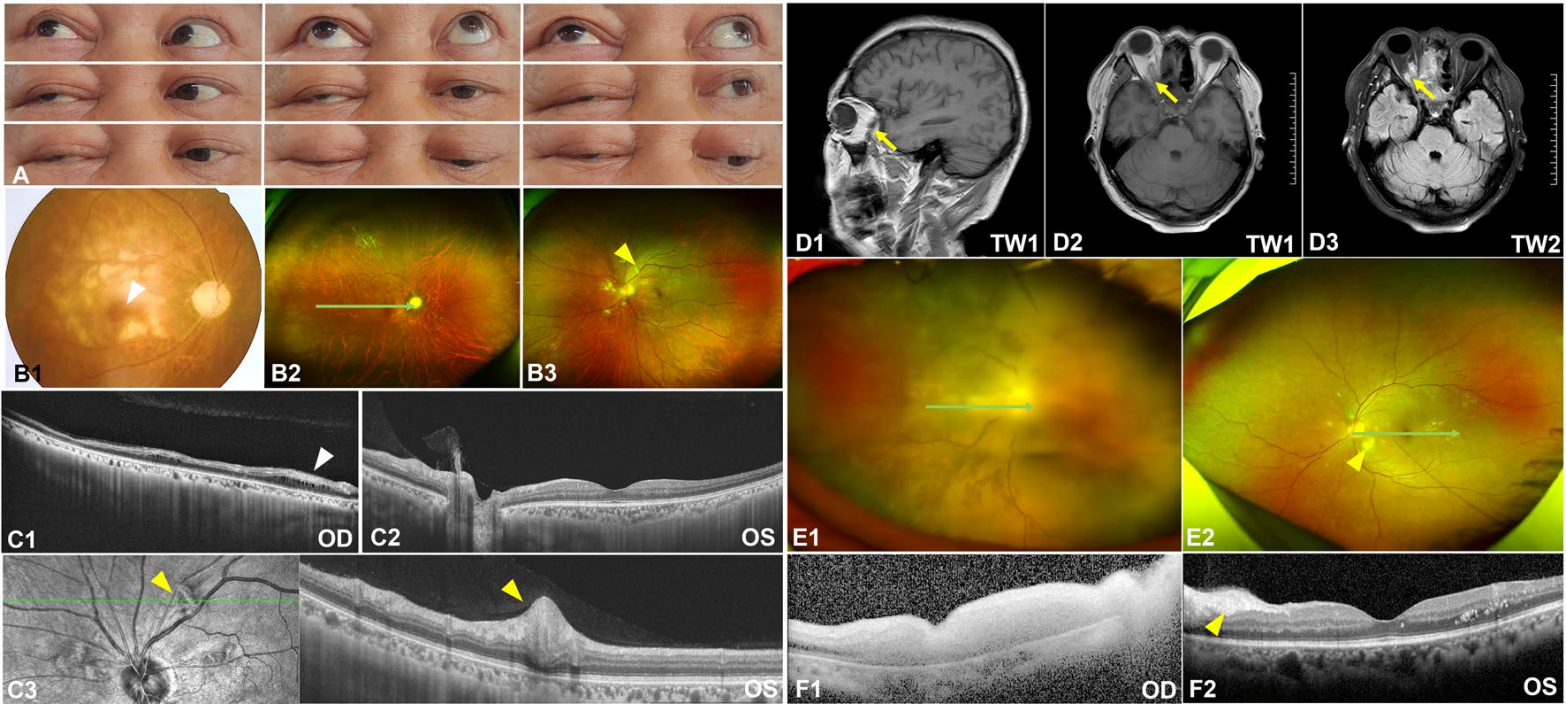
Multimodal fundus images from a 49 year-old male patient (**A**, **B**, **C**, **D**) and a 49-year-old female patient (**E**, **F**). White arrowhead showed the cherry erythema in B1, while yellow arrowhead showed the cotton-wool spots in B3 where the nerve fiber layer was swelling (C3). F1 showed the acute phase of arterial occlusion with extensive retinal edema.

A 49 year-old male patient with SARS-CoV-2-related pneumonia had recurrent fever for 20 days and underwent "intracranial hematoma drainage" because of "diabetic ketoacidosis and cerebral hemorrhage." Then, he developed swelling on the right side of the face 3 days later. Sooner afterward, he suddenly lost vision in his right eye. This patient had a history of high blood pressure and diabetes but did not take medications regularly. Due to fever and infection, his vital signs were unstable, and his right eye was not treated (Fig. 6B). Pale retinal and “cherry red spot” suggested central retinal artery occlusion (CRAO). Fungal cultures of nasal tissue in patients showed wide septum hyphae, which was characteristic of Mucormyces. This confirmed that he had ROCM. The patient's right eyeball was fixed in the upper right position and could only be slightly moved up and down instead of left and right (Fig. 6A). Unfortunately, the optic disc of the right eye was pale (Fig. 6B1). Interestingly, we observed cotton-wool spots around the optic disc of the left eye (Fig. 6B2.B3). OCT showed retinal-choroidal atrophy and interlaminar edema in his right eye (Fig. 6C1). The cotton-wool patch showed thickening of the neuroepithelium (Fig. 6C2,C3 yellow arrow head). The patient's magnetic resonance imaging (MRI) showed abnormal signals in the right orbital tip (Fig. 6D1,D2,D3).

Another 49-year-old female patient was also diagnosed with ROCM. She also had diabetic ketoacidosis, which suggested poor glycemic control. Similarly, she lost her vision in her right eye. Fundus photographs showed cotton-wool spots and hemorrhages on her left eye as well (Fig. 6E1,E2). OCT images showed the acute phase of arterial occlusion with extensive retinal edema (Fig. 6F1) and Purtscher-like retinopathy (Fig. 6F2 yellow arrow head). Blood tests showed that her white blood cells were 13.68 (reference 3.5–9.5) 10^9/L, neutrophil percentage was 83.5% (reference 40–75%), DD-dimer was as high as 10.79 (reference 0–0.3) mg/L (DDU), fibrinogen degradation products (FDP) were as high as 10.79 (reference 0–5) mg/L, and C-reactive protein (CRP) and procalcitonin (PCT) and interleukin 6 (IL-6) were more than 5 times the normal value.

Both patients did not receive ocular treatment because their vision was no light perception (NLP). In addition, three other female patients aged 77, 81, and 85 years who had ROCM and diabetes underwent eye removal.

## Discussion

There have been more than 757 million confirmed cases and 6.8 million deaths being reported worldwide since the outbreak of COVID-19 in November 2019^13^. By targeting the ACE2 receptor in human endothelial cells, SARS-CoV-2 attacks host cells through the transmembrane spike protein (S protein). Therefore, tissues with ACE2 receptors are susceptible to SARS-CoV-2 infection^14^. ACE2 is widely expressed in endothelial cells of the lung, blood vessels, heart, kidney, small intestine and other tissues and organs, with low expression in the liver and nose. These organs are vulnerable to damage following SARS-CoV-2 infection ^15,16^. Moreover, ACE2 is a major converting enzyme in the vascular protective axis of the renin-angiotensin system in the retina, and its downregulation may lead to retinal ischemia, which is related to microangiopathy, retinitis, and retinal degeneration^16–18^.

The meta-analysis conducted by Sen et al^3^ in 2021 revealed that conjunctivitis is the principal ocular manifestation following SARS-CoV-2 infection, with previous research largely focusing on individual case reports. In contrast, our research expands the scope by presenting a substantial series of cases from six medical centers, utilizing multimodal imaging to document the experiences of 90 patients. This approach provides robust evidence of ocular involvement after SARS-CoV-2 infection. Our findings underscore that in the real world, AMN is a significant and closely associated retinal lesion with after SARS-CoV-2 infection, highlighting its prevalence and clinical importance in affected patients.

Previous literature has reported retinal and choroidal manifestations that may be associated with SARS-CoV-2, as shown in Table 2. Based on the pathological mechanism, we classify these manifestations as noninfectious or infectious. Noninfectious manifestations may be related to ischemia or inflammation. RVO and RAO are common diseases in ophthalmology, and patients often have underlying diseases such as diabetes and hypertension^19,20^. Therefore, these diseases were not included in our case series as fundus lesions related to SARS-CoV-2. However, rare eye diseases such as AMN and ROCM have increased in incidence alongside the rise in SARS-CoV-2 infection rates^21,22^.

**Table 2:** Retinal and choroidal manifestations in patients after SARS-CoV-2 infection.

Type of the manifestations	Clinical findings/disease
No-infectious	Retinal vein occlusion (RVO)^23,24^ Retinal artery occlusion (RAO)^25^ Purtscher-like retinopathy^26^ Cotton-wool spots^27^ Isolated hemorrhages^28^
	Acute macular neuroretinopathy (AMN)^29^ Paracentral acute middle maculopathy (PAMM)^30^ Acute posterior multifocal placoid pigment epitheliopathy (APMPPE)^31^ Multiple evanescent white-dot syndrome (MEWDS) Serpiginous choroiditis (SC)^32^ Optic neuritis (ON)^33^ Vogt-Koyanagi-Harada syndrome-like uveitis^34^ Central serous chorioretinopathy (CSCR)^35^
Infectious	Endogenous endophthalmitis (Bacterial/Fungal)^36^ Acute retinal necrosis (ARN)^37^ Rhino-orbital-cerebral mucormycosis (ROCM)^38^

### Acute macular neuroretinopathy and Purtscher or Purtscher-like retinopathy

We observed this case series with SARS-CoV-2-related retinal and choroidal manifestations. Typically, the patient was a young woman who developed visual impairment two days following the onset of infection symptoms. As the standard, with the relationship of cause, trigger and disease, our results suggested a close relationship between the emergence of AMN or Purtscher or Purtscher-like retinopathy and SARS-CoV-2 infection.

AMN is a relatively rare disorder involving transient or permanent central or paracentral scotomas^39,40^. It is characterized by dark, reddish-brown macular lesions and corresponds precisely to visual field abnormalities^41^. AMN has been reported to occur in several different clinical settings. The majority of patients are women in their reproductive years who develop symptoms in association with oral contraceptives, hypotension, viral illness, intravitreous injection, vaccination and sympathomimetic agents (epinephrine, caffeine).^3,42–47^. Since the syndrome was initially characterized by Bos and Deutmann^49^ in 1975, the pathophysiology of AMN has been the subject of intense discussion, especially in light of the disorder's diverse causes.

Acute retinal lesions are characterized by faint retinal translucency on bio microscopy and hyperreflectivity in the outer plexiform and outer nuclear layers on OCT. FFA and ICGA do not reveal any retinal or choroidal vascular leakage, perfusion deficits or transmission defects. mfERG testing shows reduced amplitudes within the scotomatous areas^50^. Evolution of macular lesions is characterized by resolution within several days of the initial retinal translucency and hyperreflectivity, followed by the development of reddish-brown lesions that appear dark on IR and show thinning of the outer nuclear layer and attenuation or loss of the ellipsoid and interdigitation zones on OCT. Compared to FFA, ICGA and FAF, OCT and IR images display the lesions of AMN more effectively. The cSSO fundus photography used in this study is a laser with a wavelength of 820 nm; it is also an IR imaging approach in essence. Due to the destruction of the elliptical zone, the laser is absorbed by the deeper and stronger retinal epithelium, demonstrating the essence of shadow and OCT is optical coherence imaging, and the areas with mixed or dense tissue structure will show high reflection^51^. From the perspective of imaging alone, the morphology of hyper reflex in the early stage of the onset of AMN was consistent with that of the fiber of the cone and rod cells, which indicates the affected site (Fig. 1A1,A2)^41,48^. At present, the generation of AMN is mainly dominated by two theories: the inflammation-related immune theory and the vascular-related ischemia and hypoxia theory^50,52^. This needs to be discussed in combination with vascular parameters, and our research team will explore in future research.

### VKH-like uveitis and multiple evanescent white-dot syndrome (MEWDS)

VKH disease is an immune-mediated disorder characterized by bilateral uveitis frequently associated with neurological (meningeal), auditory, and integumentary symptoms. Auditory manifestations (tinnitus, hearing loss and vertigo) and others (including headache, neck and back stiffness) usually occur before or concurrently with ocular involvement^53^. A previous study linked VKH to SARS-CoV-2 Vaccines^54^. The VKH-like patients in this series responded favorably to corticosteroid therapy. The cases of VKH-like uveitis were particularly notable for their lack of typical systemic symptoms like tinnitus and skin changes, suggesting a unique, non-granulomatous panuveitis potentially triggered by COVID-19. This aligns with observations that COVID-19 may induce a distinct immune or inflammatory pathway, leading to VKH-like manifestations. Both patients showed excellent response to corticosteroid treatment without recurrence, highlighting the effectiveness of standard anti-inflammatory treatments in these atypical presentations. MWDES is related to colds and viral infections. Both patients in this study developed symptoms five days after experiencing SARS-CoV-2-related fever, which is considered a cause.

### Rhino-orbital-cerebral mucormycosis (ROCM)

ROCM can be a serious complication of severe SARS-CoV-2 infection, particularly in patients with uncontrolled diabetes. The risk factors predisposing patients to ROCM are uncontrolled diabetes, neutropenia, hematological malignancies, organ transplantation, trauma and burn, and use of immunosuppressants such as corticosteroids^38,39^. Patients were often blinded by mucormycosis invasion of the orbital apex leading to orbital apex syndrome forming retinal artery obstruction. This disease is easily misdiagnosed due to its reputation as a difficult-to-treat mold infection and its high mortality in patients with SARS-CoV-2 infection, particularly those with pulmonary disease. A careful management plan can be successful for rhino-orbital cerebral disease if there is early diagnosis of infection and control of infection^55^.

In conclusion, the retinal and choroidal conditions after SARS-CoV-2 infection are diverse, including AMN, MEWDS, VKH-like uveitis, and ROCM. Multimodal imaging may be used to evaluate the lesions from the anatomical and functional levels, and an appropriate examination with multimodal imaging is beneficial for patient management and follow-up.

## Data Availability

The data that support the findings of this study are available from the corresponding author upon reasonable request.

## References

[1] Sharma A, Tiwari S, Deb MK, Marty JL (2020). Severe acute respiratory syndrome coronavirus-2 (SARS-CoV-2): A global pandemic and treatment strategies. Int. J. Antimicrob. Agents.

[2] Costa IF (2021). Ocular findings among patients surviving COVID-19. Sci. Rep..

[3] Sen M (2021). COVID-19 and eye: A review of ophthalmic manifestations of COVID-19 Indian. J. Ophthalmol..

[4] Karampelas M, Dalamaga M, Karampela I (2020). Does COVID-19 involve the retina?. Ophthalmol. Ther..

[5] Harvey WT (2021). SARS-CoV-2 variants, spike mutations and immune escape. Nat. Rev. Microbiol..

[6] Aleem, A., Akbar Samad, A. B. & Slenker, A. K. Emerging Variants of SARS-CoV-2 And Novel Therapeutics Against Coronavirus (COVID-19). *StatPearls* (2022). 34033342

[7] Jevnikar K (2021). An update on COVID-19 related ophthalmic manifestations. Ocul. Immunol. Inflamm..

[8] Invernizzi A (2020). Retinal findings in patients with COVID-19: Results from the SERPICO-19 study. EClinicalMedicine.

[9] Zhang Y, Stewart JM (2021). Retinal and choroidal manifestations of COVID-19. Curr. Opin. Ophthalmol..

[10] McGrath OE, Aslam TM (2022). Use of imaging technology to assess the effect of COVID-19 on retinal tissues: A systematic review. Ophthalmol. Ther..

[11] Pan Y (2023). Characterisation of SARS-CoV-2 variants in Beijing during 2022: an epidemiological and phylogenetic analysis. Lancet.

[12] Read RW (2021). Classification criteria for vogt-koyanagi-harada disease. Am. J. Ophthalmol..

[13] World Health Organization. https://www.who.int/europe/emergencies/situations/covid-19 (Updated 2023.02.05).

[14] Jackson CB (2022). Mechanisms of SARS-CoV-2 entry into cells. Nat. Rev. Mol. Cell Biol..

[15] Guney C, Akar F (2021). Epithelial and endothelial expressions of ACE2: SARS-CoV-2 entry routes. J. Pharm. Pharm. Sci..

[16] Pagliaro P (2022). Angiotensin-converting enzyme 2: a key enzyme in key organs. J. Cardiovasc. Med. (Hagerstown).

[17] Yener AU (2021). COVID-19 and the eye: Ocular manifestations, treatment and protection measures. Ocul. Immunol. Inflamm..

[18] Lumbers ER (2022). The interacting physiology of COVID-19 and the renin-angiotensin-aldosterone system: Key agents for treatment. Pharmacol. Res. Perspect..

[19] Hayreh SS, Podhajsky PA, Zimmerman MB (2009). Retinal artery occlusion: Associated systemic and ophthalmic abnormalities. Ophthalmology.

[20] Modjtahedi BS, Do D, Shaw J (2022). Correspondence regarding changes in the incidence of retinal vascular occlusions after COVID-19 diagnosis-reply. JAMA Ophthalmol..

[21] Azar G (2021). Did the COVID-19 pandemic increase the incidence of acute macular neuroretinopathy?. J. Clin. Med..

[22] Nehara HR (2022). Coronavirus disease, diabetes and glucocorticoid a terrible trio for invasive mucormycosis: An observational study from Northwest Rajasthan. J. Assoc. Phys. India.

[23] Nourinia R (2021). Branch retinal vein occlusion after COVID-19. J. Fr. Ophtalmol..

[24] Cuadros Sanchez C (2022). Central retinal vein occlusion presumably associated with lupus anticoagulant induced by SARSCoV-2. Ocul. Immunol. Inflamm..

[25] Au SCL, Ko CKL (2021). Central retinal artery occlusion in patients with COVID-19: Imaging for underlying causes. Radiology.

[26] Thatcher MD, Wu LZ, Varma R (2023). Bilateral purtscher-like retinopathy associated with COVID-19 infection. JAMA Ophthalmol..

[27] Chan AX, Ritter M, Bakhoum MF (2021). Bilateral cotton wool spots after ambulatory COVID-19. Int. J. Infect. Dis..

[28] Pereira LA (2022). Retinal findings in hospitalised patients with severe COVID-19. Br. J. Ophthalmol..

[29] Giacuzzo C, Eandi CM, Kawasaki A (2022). Bilateral acute macular neuroretinopathy following COVID-19 infection. Acta Ophthalmol..

[30] Castro CS (2022). Paracentral acute middle maculopathy after COVID-19 disease: Multimodal evaluation. Retin. Cases Brief Rep..

[31] Fischer NA, Wann RC, Crosson JN (2023). Acute posterior multifocal placoid pigment epitheliopathy following COVID-19 infection. Am. J. Ophthalmol. Case Rep..

[32] Providencia J (2022). Serpiginous choroiditis presenting after SARS-CoV-2infection: A new immunological trigger?. Eur. J. Ophthalmol..

[33] Landecho MF (2021). COVID-19 retinal microangiopathy as an in vivo biomarker of systemic vascular disease?. J. Intern. Med..

[34] Yepez JB (2021). Vogt-Koyanagi-Harada disease following COVID-19 infection. Case Rep. Ophthalmol..

[35] Mohd-Alif WM (2022). Bilateral and multiple central serous chorioretinopathy following COVID-19 infection: A case report and literature review. Cureus.

[36] Agarwal M (2021). Endogenous endophthalmitis a complication of COVID-19 pandemic: A case series. Ocul. Immunol. Inflamm..

[37] Nishiyama T (2022). Acute retinal necrosis in a patient on immunosuppressive treatment for COVID-19 pneumonia: A case report. BMC Ophthalmol..

[38] Kaur R, Khan B, Sharma A (2021). Optical coherence tomography of retinal artery occlusion associated with mucormycosis and COVID-19. JAMA Ophthalmol..

[39] Dubey S (2021). COVID-19 associated rhino-orbital-cerebral mucormycosis: An observational study from Eastern India, with special emphasis on neurological spectrum. Diabetes Metab. Syndr..

[40] Hufendiek K (2018). Classification and characterization of acute macular neuroretinopathy with spectral domain optical coherence tomography. Int. Ophthalmol..

[41] Fawzi AA (2012). Acute macular neuroretinopathy: long-term insights revealed by multimodal imaging. Retina.

[42] Fekri S (2023). Acute macular neuroretinopathy and COVID-19 vaccination: Case report and literature review. J. Fr. Ophtalmol..

[43] Powers JH (2021). Multimodal imaging of type 2 acute macular neuroretinopathy in a young woman. Digit. J. Ophthalmol..

[44] Gupta N (2019). Acute macular neuroretinopathy (AMN) related to energy drink consumption. BMJ Case Rep..

[45] Ashfaq I (2021). Acute macular neuroretinopathy associated with acute influenza virus infection. Ocul. Immunol. Inflamm..

[46] Dutta Majumder P, Agarwal A (2023). Acute macular neuroretinopathy and paracentral acute middle maculopathy during SARS-CoV-2 infection and vaccination. Vaccines (Basel).

[47] Radwan LM (2022). Acute macular neuroretinopathy associated with intravitreal anti-VEGF injection: A case report. Am. J. Ophthalmol. Case Rep..

[48] Guardiola GA (2022). Acute macular neuroretinopathy in dengue virus serotype 1. Am. J. Ophthalmol. Case Rep..

[49] Bos PJ, Deutman AF (1975). Acute macular neuroretinopathy. Am. J. Ophthalmol..

[50] Bhavsar KV (2016). Acute macular neuroretinopathy: A comprehensive review of the literature. Surv. Ophthalmol..

[51] Lains I (2021). Retinal applications of swept source optical coherence tomography (OCT) and optical coherence tomography angiography (OCTA). Prog. Retin. Eye Res..

[52] Kulikov AN (2020). Retinal microvasculature alteration in paracentral acute middle maculopathy and acute macular neuroretinopathy: A quantitative optical coherence tomography angiography study. Retin. Cases Brief Rep..

[53] Du L, Kijlstra A, Yang P (2016). Vogt-Koyanagi-Harada disease: Novel insights into pathophysiology, diagnosis and treatment. Prog. Retin. Eye Res..

[54] Chen X (2022). Ocular adverse events after inactivated COVID-19 vaccination in Xiamen. Vaccines (Basel).

[55] Hoenigl M (2022). The emergence of COVID-19 associated mucormycosis: a review of cases from 18 countries. Lancet Microbe.

